# Velocity- and Power-Oriented Force–Velocity Characteristics Differentiate Competitive Olympic-Style Weightlifting Performance

**DOI:** 10.3390/jfmk11020147

**Published:** 2026-04-01

**Authors:** Athanasios Tsoukos, Theodoros Iakovidis, Sofia Georgopoulou, Gregory C. Bogdanis

**Affiliations:** School of Physical Education and Sport Science, National and Kapodistrian University of Athens, 172 37 Athens, Greece; thodoris.iakovidis91@gmail.com (T.I.); sophge229@gmail.com (S.G.); gbogdanis@phed.uoa.gr (G.C.B.)

**Keywords:** front squat, clean performance, 1-RM, force–velocity profile, countermovement jump, allometric scaling, relative strength

## Abstract

**Background**: This study examined the associations between dynamic maximum strength (front squat [FS] and clean [CL]), lower-limb vertical force–velocity (F–V) profile characteristics, and both absolute and scaled measures of competitive weightlifting performance in trained weightlifters. **Methods**: Fourteen competitive male weightlifters (age: 27.6 ± 4.2 years; height: 1.74 ± 0.05 m; body mass: 85.1 ± 6.7 kg; body fat: 11.7 ± 2.8%) completed three testing sessions separated by 48–72 h, including 1-RM assessment in the FS and CL, as well as vertical countermovement jump trials to determine individual force–velocity profile parameters (F_0_, V_0_, and Pmax). Official competition results obtained within the same competitive season were recorded for the snatch (SN), clean and jerk (C&J), total (TOT), and Sinclair score. Participants were additionally divided into higher and moderate jump performance groups using a median split of unloaded countermovement jump height. **Results**: Very strong correlations were found between 1-RM strength (FS and CL) and weightlifting performance, with CL showing the strongest associations with SN (r = 0.82), C&J (r = 0.93), and TOT (r = 0.94). Among F–V parameters, V_0_ and Pmax were significantly associated with competitive outcomes (r = 0.63–0.70), whereas F_0_ was not. V_0_ was significantly associated with SN (r = 0.69), C&J (r = 0.63), and TOT (r = 0.70), while F_0_ showed trivial-to-small associations (r = 0.08–0.28). When participants were divided using a median split of CMJ height, higher jumpers exhibited greater V_0_ (3.02 ± 0.30 vs. 2.61 ± 0.23 m·s^−1^, *p* = 0.014, g = 1.4) and relative Pmax (32.44 ± 2.65 vs. 27.28 ± 1.06 W·kg^−1^, *p* = 0.001, g = 2.4), despite similar F_0_ (*p* = 0.67). Higher jumpers also demonstrated superior SN (*p* = 0.016, g = 1.4), C&J (*p* = 0.041, g = 1.1), TOT (*p* = 0.018, g = 1.4), and Sinclair scores (*p* = 0.001, g = 2.1). **Conclusions**: In trained weightlifters, performance was strongly associated with maximal strength, while velocity- and power-oriented characteristics (V_0_ and Pmax) were also associated with performance outcomes. In contrast, F_0_ showed no meaningful associations with performance within this sample. These findings suggest that, among already strength-trained athletes, the ability to express force at higher contraction velocities may be associated with differences in competitive performance.

## 1. Introduction

Olympic-style weightlifting includes two competition lifts: the snatch (SN) and the clean and jerk (C&J), from which numerous training derivatives are developed. In formal competition, athletes are ranked within bodyweight categories according to the combined total of their best successful attempt in each lift [1]. Because performance is influenced by body mass, competitive outcomes are often normalized using the Sinclair coefficient (SIN) or alternative allometric scaling methods to allow meaningful comparisons across athletes of different classes or sexes [2].

From a strength and conditioning perspective, coaches frequently incorporate strength- and power-oriented weightlifting derivatives, such as the front squat (FS) and the clean or power clean [3,4,5,6], to enhance Olympic weightlifting performance [4,7,8,9]. These exercises are used to develop lower-limb strength and explosive pulling capacity [5,9,10], neuromuscular qualities that underpin rapid force production and effective barbell acceleration during the SN and C&J [2,4,11,12,13].

The rationale for their widespread implementation is supported by previous findings demonstrating significant relationships between performance in these derivatives and the competitive Olympic weightlifting [2,4]. In particular, FS strength has been reported to show very strong correlations with snatch, clean and jerk, and total performance (r = 0.85 to 0.88), supporting its role as a key indicator of lower-limb maximal strength in weightlifters [7]. Similarly, recent evidence has highlighted the importance of neuromuscular force characteristics assessed through pull-specific isometric tests [4,14,15]. For example, isometric mid-thigh pull (IMTP) peak force has been shown to explain a substantial proportion of the variance in total, snatch, and clean and jerk performance in international male and female weightlifters [14,15], in cross-sectional analyses, while changes in IMTP peak force predicted longitudinal changes in competition performance [14]. However, despite the strong predictive value of maximal force capacity assessed under both dynamic (e.g., FS) and isometric (IMTP) conditions, limited research has directly examined the relationship between dynamic 1-RM clean (CL) performance and competitive Olympic weightlifting outcomes. Consequently, it remains unclear whether maximal dynamic CL strength demonstrates associations with SN, C&J and TOT performance, particularly when scaled performance indicators are considered.

Beyond maximal force capacity, successful weightlifting performance depends on the interaction between force and velocity characteristics [16,17,18]. The force–velocity (F–V) profile provides insight into an athlete’s mechanical capacities, including theoretical maximal force (F_0_), theoretical maximal velocity (V_0_), and maximal power output (Pmax) [19,20,21]. While the F–V profile has been extensively investigated in sprinting and jumping tasks, its relationship with competitive weightlifting performance, particularly in conjunction with strength derivatives such as the FS and CL, remains insufficiently explored. Given that SN and C&J require rapid force expression against substantial external loads, velocity-oriented neuromuscular qualities may meaningfully differentiate performance among athletes who already possess high maximal strength.

Considering the above and the lack of research linking the force–velocity profile characteristics to competitive weightlifting performance, the purpose of the present study was to examine the associations between front squat strength, clean performance, lower-limb vertical force–velocity profile parameters with both absolute and scaled measures of competitive weightlifting performance (snatch, clean and jerk, total, Sinclair total, and allometrically adjusted results) in competitive weightlifters. We hypothesized that (i) FS and CL 1-RM would show very strong associations with competitive outcomes, and (ii) velocity- and power-related F–V parameters (V_0_ and Pmax), but not F_0_, would further associate with and differentiate competitive performance beyond maximal strength in this trained sample.

## 2. Materials and Methods

### 2.1. Participants

Fourteen male competitive weightlifters volunteered to participate in the study (age: 27.6 ± 4.2 years; height: 1.74 ± 0.05 m; body mass: 85.1 ± 6.7 kg; body fat: 11.7 ± 2.8%). Participants were recruited through direct personal invitation from competitive Olympic-style weightlifting clubs. All participants had a minimum of five years of structured Olympic-style weightlifting training experience and regularly competed at national and/or international levels. Inclusion criteria were: (a) at least five years of consistent Olympic-style weightlifting training, (b) absence of musculoskeletal injury within the previous 12 months, and (c) no self-reported use of performance-enhancing substances. Body fat percentage was assessed using bioelectrical impedance analysis (BIA) (MC-780MA Tanita, Tokyo, Japan) under standardized conditions [22]. Prior to participation, all athletes received a detailed explanation of the study procedures, potential risks, and their right to withdraw at any time without penalty. Written informed consent was obtained from each participant. The study was approved by the local Institutional Review Board (Approval no. 1821/19 June 2025) and was conducted in accordance with the ethical standards of the Declaration of Helsinki of 1964, revised 2013.

### 2.2. Research Design

A cross-sectional observational design was used to investigate the relationships between front squat strength (FS), clean (CL) performance, lower-limb force–velocity (F–V) profile parameters, and competitive Olympic weightlifting outcomes. Participants completed three standardized testing sessions conducted at a weightlifting training facility, followed by participation in an official weightlifting competition. Competition results were collected from an official competition conducted within the same competitive season and within approximately 2 months of the neuromuscular testing sessions, during the pre-competitive and competitive phases of the season. During the first session, participants performed a one-repetition maximum test in the front squat. In the second session, 1-RM performance in the clean was assessed. During the third session, athletes completed vertical jump trials to determine the individual force–velocity profile parameters. All sessions were separated by at least 48–72 h to minimize the effects of residual fatigue.

### 2.3. Maximum Strength (1-RM) in the Front Squat and Clean

Maximum dynamic strength (one-repetition maximum: 1-RM) in the FS and CL was assessed on a regulation Olympic weightlifting platform using standardized Olympic barbells and calibrated weight plates in accordance with the testing guidelines outlined by the National Strength and Conditioning Association (NSCA) [3,23,24,25]. Prior to testing, participants completed a structured warm-up consisting of dynamic mobility exercises (e.g., leg swings in sagittal and frontal planes, walking lunges, Cossack squats, ankle rockers, and inchworm-to-push-up drills), followed by neuromuscular activation and joint preparation exercises (e.g., squat pulses, band-assisted front rack pulses, and single-leg Romanian deadlifts). This was followed by low-amplitude plyometric activities (pogo-type jumps) and 2–3 maximal vertical jumps performed with full recovery between efforts to enhance neural readiness. Finally, athletes performed progressive barbell-specific movements, including empty-bar technical drills and submaximal preparatory sets. Following the warm-up, the load was progressively increased in successive attempts [26,27] until the participant achieved the maximal load that could be lifted once with proper technical execution [28,29]. An unsuccessful attempt was defined as the inability to complete the lift through the full range of motion or failure to maintain the required technical standards. Testing was terminated when a lift was deemed unsuccessful despite verbal encouragement. A rest interval of three minutes was provided between attempts to ensure adequate recovery and minimize fatigue-related performance decrements. The highest successfully completed lift was recorded as the participant’s 1-RM. To ensure participant safety and standardized testing conditions, two experienced spotters were present throughout the assessment. Both were certified strength and conditioning coaches with extensive competitive experience in Olympic weightlifting, providing technical supervision and standardized verbal encouragement during maximal attempts.

### 2.4. Force–Velocity Profile (F–V)

The assessment of the force–velocity profile was conducted during the third testing session using countermovement jumps performed with progressively increasing external loads. Countermovement jump (CMJ) trials were recorded using a smartphone operating at 240 frames per second (fps) [21,30,31], and video analysis was performed using the MyJump Lab 3 application, which has been previously validated for the assessment of jump performance [30,32,33]. A standardized rest interval of two minutes was provided between attempts to minimize the influence of fatigue and ensure measurement accuracy. The collected data across loading conditions were subsequently used to determine individual F–V profile parameters, including theoretical maximal force (F_0_), theoretical maximal velocity (V_0_), and maximal power output (Pmax) [31,34,35,36]. For the CMJ, participants stood with their feet shoulder-width apart and hands on their hips to eliminate arm swing. They lowered to a knee angle of approximately 90° and then jumped as high as possible [28,37]. In accordance with established force–velocity profiling procedures, multiple loading conditions (including unloaded jumps) were used to ensure an adequate spread of data points across the force–velocity spectrum. External load was applied using a standard Olympic barbell positioned across the upper back (back squat position). Participants performed jumps under progressively increasing external loads (0, 20, 40, 60, 80, 100, and up to 120 kg), provided they were able to maintain proper technique. In cases where jump height was markedly reduced (≈10–14 cm), no further increases in load were applied [31]. Two attempts were performed at each load, and the best performance was retained for subsequent analysis.

### 2.5. Competition Performance Assessment

Competitive performance data were obtained from an official Olympic weightlifting competition conducted under the regulations of the International Weightlifting Federation [1]. Competition results were collected within the same competitive season and in close temporal proximity to the neuromuscular testing sessions. Athletes performed the snatch and clean and jerk according to standard competition rules, with three attempts allowed per lift [1]. The highest successfully completed attempt in each lift was recorded, and total performance (TOT) was calculated as the sum of the best snatch and clean and jerk results [1]. Sinclair coefficients were applied to normalize performance relative to body mass, and Sinclair total (SIN) was calculated according to the official IWF formula for the corresponding competition cycle [38]. Sinclair scores were calculated using competition-day body mass, which was, on average, ~1.6 kg lower than laboratory-assessed body mass. Allometric scaling procedures were additionally applied to account for body mass differences where appropriate. Specifically, variables were scaled according to body mass raised to the power of 0.67 (i.e., variable/body mass^0.67^), as commonly used in strength and power research [39].

### 2.6. Statistical Analysis

Statistical analyses were conducted using STATISTICA (v.12.0; StatSoft Inc., Tulsa, OK, USA). Data are presented as means ± standard deviations (SD). Pearson’s product–moment correlation coefficients (r) were calculated to examine the relationships between neuromuscular and competitive performance variables. The magnitude of correlations was interpreted as small (0.10–0.29), moderate (0.30–0.49), large (0.50–0.69), or very large (≥0.70). For exploratory and practical comparison purposes, participants were divided into higher and moderate jump performance groups using a median split of unloaded countermovement jump height, given the relevance of lower-limb explosive performance to Olympic-style weightlifting [14,16,40,41]. This subgrouping approach was not intended as a definitive classification method, but rather as a simple way to compare athletes with relatively higher versus moderate jump performance within the present sample. Independent-samples *t*-tests were performed to assess between-group differences. Effect sizes were calculated using Hedges’ g and interpreted as small (<0.30), moderate (0.30–0.80), or large (>0.80). Statistical significance was set at *p* < 0.05. Given the exploratory nature of the analysis and the number of bivariate associations, correlation magnitudes and consistency of patterns across outcomes were emphasized alongside *p*-values. Assumptions for parametric analyses were assessed prior to statistical testing. Normality of the data was evaluated using the Shapiro–Wilk test and visual inspection of histograms, and all variables met the assumption of normality (*p* > 0.05). Linearity and potential outliers were examined through visual inspection of scatterplots, and no substantial deviations from linearity or influential outliers were observed. Given the relatively small sample size, Spearman rank-order correlations were additionally performed as a robustness check, yielding a similar pattern of results to Pearson correlations.

## 3. Results

### 3.1. Descriptive Characteristics of the Sample

Descriptive statistics of anthropometric characteristics, maximal strength performance, force–velocity profile parameters, and competitive weightlifting outcomes are presented in Table 1.

Overall, the values indicate a sample of trained competitive weightlifters with performance levels consistent with national-level standards and well-developed lower-limb strength and power characteristics.

### 3.2. Correlation Analysis

Pearson’s correlation analysis revealed very strong associations between maximal strength variables and competitive performance. In particular, 1-RM clean demonstrated the strongest relationships with snatch (r = 0.82, 95% CI: 0.51 to 0.94), clean and jerk (r = 0.93, 95% CI: 0.79 to 0.98), and total performance (r = 0.94, 95% CI: 0.82 to 0.98), while similarly strong correlations were observed for 1-RM front squat (r = 0.72–0.82 across performance measures; 95% CI: 0.31 to 0.94). These findings highlight the prominent contribution of dynamic maximal strength to competitive weightlifting outcomes. The results of the correlation analysis are presented in Table 2.

**Table 2:** Pearson correlations between neuromuscular variables and competitive weightlifting performance.

Variable	Snatch	Clean & Jerk	Total	Sinclair
1-RM Front Squat (kg)	0.72 **	0.81 **	0.82 **	0.80 **
1-RM Clean (kg)	0.82 **	0.93 **	0.94 **	0.81 **
CMJ (cm)	0.55 *	0.54 *	0.58 *	0.62 *
F_0_ (N)	0.08	0.28	0.20	0.10
V_0_ (m·s^−1^)	0.69 **	0.63 *	0.70 **	0.49
Pmax (W)	0.64 *	0.67 **	0.70 **	0.47
Pmax (W·kg^−1^)	0.53 *	0.46	0.52	0.60 *
Pmax (W∙kg^−0.67^)	0.60 *	0.57 *	0.62 *	0.58 *

Bold values indicate statistically significant correlations; **: *p* ≤ 0.01; *: *p* ≤ 0.05.

Among force–velocity profile parameters, V_0_ exhibited significant correlations with snatch (r = 0.69, *p* < 0.01, 95% CI: 0.25 to 0.89), clean and jerk (r = 0.63, *p* < 0.05, 95% CI: 0.15 to 0.87), and total performance (r = 0.70, *p* < 0.01, 95% CI: 0.27 to 0.90), whereas F_0_ showed no meaningful associations with any competitive variable (r = 0.08–0.28, 95% CI: −0.47 to 0.71). Maximal power output (Pmax, absolute) was moderately to strongly associated with snatch (r = 0.64, 95% CI: 0.17 to 0.87), clean and jerk (r = 0.67, 95% CI: 0.22 to 0.89), and total (r = 0.70, 95% CI: 0.27 to 0.90), indicating that lower-limb power production capacity contributes to competitive performance. When performance was normalized using the Sinclair coefficient, correlations with V_0_ (r = 0.49, *p* = 0.072, 95% CI: −0.05 to 0.81) and absolute Pmax (r = 0.47, *p* = 0.088, 95% CI: −0.08 to 0.80) approached, but did not reach, statistical significance. Similarly, relative Pmax (W·kg^−1^) demonstrated a significant association with snatch (r = 0.60, 95% CI: 0.10 to 0.86) but did not reach significance with clean and jerk and total performance (*p* values ranging from 0.056 to 0.099). On the other hand, Pmax allometric scaling showed significant associations with all variables. These findings suggest that the magnitude of the association between lower-limb power characteristics and competitive outcomes may be influenced by the scaling approach applied to performance variables.

### 3.3. Relationships Between Front Squat, Clean Performance, and Lower-Limb Force–Velocity Characteristics

Pearson’s correlation analysis showed a very strong relationship between FS and CL performance (r = 0.86, *p* < 0.01, 95% CI: 0.60 to 0.95), indicating substantial overlap in dynamic maximal strength capacity between the two lifts (Table 3). Regarding force–velocity profile parameters, theoretical maximal force (F_0_) demonstrated weak and non-significant relationships with both front squat (r = 0.19, 95% CI: −0.38 to 0.65) and clean performance (r = 0.43, 95% CI: −0.13 to 0.78). In contrast, theoretical maximal velocity (V_0_) was moderately and significantly correlated with front squat (r = 0.64, *p* ≤ 0.05, 95% CI: 0.17 to 0.87) and clean performance (r = 0.62, *p* ≤ 0.05, 95% CI: 0.13 to 0.87). Maximal power output exhibited consistent and meaningful associations with both strength exercises (FS and CL). Absolute Pmax (W) correlated with front squat (r = 0.65, *p* ≤ 0.05, 95% CI: 0.18 to 0.88) and clean (r = 0.72, *p* < 0.01, 95% CI: 0.31 to 0.90). Similarly, relative Pmax (W·kg^−1^) was significantly related to front squat (r = 0.65, *p* ≤ 0.05, 95% CI: 0.18 to 0.88) and clean performance (r = 0.54, *p* ≤ 0.05, 95% CI: 0.01 to 0.84). Allometrically scaled Pmax (W·kg^0.67^) demonstrated significant and higher correlations than relative Pmax, with front squat (r = 0.68, *p* < 0.01, 95% CI: 0.24 to 0.90) and clean (r = 0.64, *p* < 0.01, 95% CI: 0.16 to 0.88), suggesting that appropriate scaling may slightly strengthen the observed associations. Countermovement jump height (CMJ) was also significantly correlated with both front squat (r = 0.67, *p* < 0.01, 95% CI: 0.21 to 0.89) and clean performance (r = 0.59, *p* ≤ 0.05, 95% CI: 0.07 to 0.86), further supporting the link between lower-limb explosive capacity and dynamic maximal strength performance.

### 3.4. Differences Between Higher and Moderate Jump Performance Groups

Using a median split of unloaded countermovement jump (CMJ) height, the sample was divided into higher and moderate jump performance groups (*n* = 7 per group). As shown in Table 4, no significant differences were observed between groups in anthropometric characteristics, including height (*p* = 0.841), body mass (*p* = 0.506), and body fat percentage (*p* = 0.709), with small effect sizes (g = 0.24–0.47). In contrast, significant between-group differences were evident in competitive weightlifting performance. High jumpers demonstrated superior performance in the snatch (*p* = 0.016, g = 1.40), clean and jerk (*p* = 0.041, g = 1.14), and total (*p* = 0.018, g = 1.37), all with large effect sizes. The difference was even more pronounced for the Sinclair score (*p* = 0.001, g = 2.13), indicating a very large effect.

**Figure 1:**
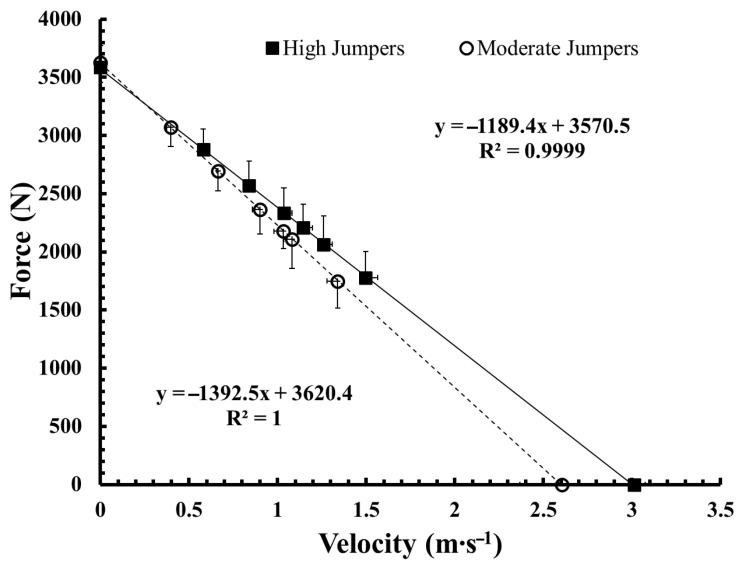
Force–velocity profiles of high and moderate jump performance groups. Force–velocity relationships obtained from the loaded countermovement jump (CMJ) test for the high jumpers (■, *n* = 7) and moderate jumpers (○, *n* = 7) groups. Groups were formed using a median split of unloaded CMJ height. Data points represent mean values across loads, with vertical error bars indicating standard deviation. Linear regression lines describe the individual group force–velocity relationships. High jumpers demonstrated greater theoretical maximal velocity (V_0_) and relative maximal power output (Pmax), whereas no differences were observed in theoretical maximal force (F_0_) between groups. Velocity is expressed in m·s^−1^ and force in Newtons. The solid line represents the force–velocity regression of the high jumpers, while the dashed line represents the force–velocity regression of the moderate jumpers.

Regarding maximal strength, high jumpers exhibited significantly greater 1-RM front squat (*p* = 0.001, g = 2.06) and 1-RM clean performance (*p* = 0.045, g = 1.12), again with very large effect sizes. From a mechanical perspective, high jumpers demonstrated significantly greater theoretical maximal velocity (V_0_; *p* = 0.014, g = 1.44), relative maximal power output (Pmax W·kg^−1^; *p* = 0.001, g = 2.40), and allometrically scaled Pmax (*p* = 0.002, g = 1.91). As expected, CMJ performance itself differed markedly between groups (*p* < 0.001, g = 2.41). Overall, while body size characteristics were comparable between groups, higher jump performance was associated with substantially greater maximal strength, relative power output, and competitive weightlifting performance, with consistently large effect sizes across neuromuscular and competition-related variables. No significant differences were observed between groups in theoretical maximal force (F_0_) expressed either in absolute or relative terms (*p* > 0.05), despite the clear differences in velocity- and power-related parameters. These results are illustrated in Figure 1.

## 4. Discussion

The main finding of the present study was that dynamic maximal strength—particularly 1-RM clean—was very strongly associated with competitive Olympic weightlifting performance. Moreover, within this level of athletes, velocity- and power-oriented force–velocity characteristics were associated with higher performance levels in this sample, whereas force-oriented parameters (F_0_) showed no meaningful associations. Practically, these data suggest that jump-derived velocity and power qualities may reflect performance-relevant neuromuscular characteristics among athletes who already possess high maximal strength.

The present study highlights the major role of maximal dynamic strength as a primary factor associated with competitive weightlifting performance. In the current sample, 1-RM CL and FS performance showed very strong associations with SN, C&J, and total performance, supporting the concept that lower-body multi-joint strength capacity underpins competitive success. These results are consistent with previous studies reporting large to nearly perfect correlations between squat-based strength measures and weightlifting performance [2,7]. For example, Stone et al. (2005) reported very strong relationships between maximum squat strength and weightlifting performance, demonstrating relationships of r = 0.94 (men) and r = 0.79 (women) with the snatch, and r = 0.95 (men) and r = 0.86 (women) with the clean [2]. When performance is expressed relative to body mass (kg∙kg^−1^) or normalized using the Sinclair formula, allometric scaling, or height-adjusted scaling, the associations between squat strength and weightlifting performance remained consistently large across normalization methods [2]. Similar findings have been reported in competitive lifters, where both dynamic squat strength [7] and isometric pull strength [15] demonstrated large to nearly perfect correlations with snatch, clean and jerk, and total performance.

While maximal strength provides the structural foundation for weightlifting performance, the findings of the present study are in agreement with recent work, suggesting that force–velocity profile characteristics offer additional insight into mechanical determinants of explosive tasks. A recent systematic review reported that F–V parameters derived from vertical profiling discriminate between athletes of different performance levels and are significantly associated with sport performance outcomes [34]. Specifically, maximal power output (Pmax) has consistently demonstrated the strongest relationships with ballistic performance measures [34]. In weightlifting athletes, countermovement jump peak power and isometric peak force have been shown to predict a substantial proportion of the variance in snatch (r = 0.88, *p* < 0.01) and clean and jerk performance (r = 0.76, *p* < 0.01) [14], supporting the relevance of neuromuscular power characteristics. However, limited research has directly examined the association between F–V profile parameters and competitive weightlifting outcomes. Previous work indicates that maximal strength levels are related to Pmax and broader F–V characteristics [18], and that the barbell-derived force–velocity relationship parameters, including Pmax, may track changes in snatch performance over time [17]. In the present study, theoretical maximal velocity (V_0_) was significantly associated with competitive performance (e.g., snatch, clean and jerk, and total), while theoretical maximal force (F_0_) did not demonstrate meaningful relationships. Possibly, the relatively homogeneous and strength-trained nature of the current sample may have limited the variability of force-oriented parameters, potentially attenuating correlation magnitudes due to restriction of range. Nevertheless, these findings suggest that within already strong lifters, velocity-oriented characteristics may be more closely associated with performance level than maximal force capacity in this sample. These findings are in line with previous research demonstrating that neuromuscular characteristics related to rapid force production are associated with weightlifting performance. For example, Zaras et al. (2021) reported significant relationships between isometric rate of force development (RFD) and both snatch and clean and jerk performance in elite weightlifters, highlighting the importance of rapid force expression in weightlifting tasks [42]. This aligns with evidence that velocity-dominant profiles may exert a stronger influence on explosive performance variability [43].

When the participants were divided into “higher” and “moderate” jumpers, we observed substantial differences between groups in maximal strength, velocity-oriented F–V parameters, and competitive weightlifting performance, although there were no differences in anthropometric characteristics. These findings further extend and support the correlation findings and provide a more applied performance model. High jumpers showed greater FS and CL 1-RM values, as well as superior SN, C&J, total, and Sinclair scores, with consistently very large effect sizes. From a mechanical standpoint, the most notable differences were observed in theoretical maximal velocity (V_0_) and power-related parameters (Pmax expressed relative and allometrically), whereas theoretical maximal force (F_0_) did not differ between groups. This pattern suggests that once a sufficient force threshold has been achieved—as expected in well-trained weightlifters- performance differences may be related to the ability to express force rapidly, rather than being explained solely by maximal force capacity. These findings are consistent with previous work indicating strong associations between countermovement jump peak power and weightlifting performance [14,44], as well as results that CMJ performance is a strong predictor of Sinclair scores in female weightlifters [41]. Furthermore, kinematic and kinetic analyses have demonstrated significant relationships between Olympic weightlifting and the vertical jump performance, particularly in lower-limb force production and propulsion characteristics [45,46]. These findings support the notion that vertical jump performance reflects explosive neuromuscular qualities that are biomechanically similar to competitive weightlifting. Collectively, the present study suggests that in trained weightlifters, maximum force production appears to be an important prerequisite, but may not fully explain differences in competitive performance among athletes. Instead, the ability to express force at higher contraction velocities may represent an important factor associated with performance differences between athletes of comparable body size and overall strength capacity.

In the present study, F_0_ did not differ between high and moderate jumpers, whereas 1-RM performance in the FS, CL, and competitive lifts was significantly greater in high jumpers. This apparent discrepancy can be explained in light of the findings of Riviere et al. (2017), who examined the position of the one-repetition maximum (1-RM) squat on the force–velocity (F–V) relationship derived from squat jumps [47]. The authors demonstrated that the 1-RM point is aligned with the linear F–V relationship but is located below F_0_, typically corresponding to ~90% of F_0_, and occurring at a non-negligible velocity (~0.22 m∙s^−1^) [47]. Importantly, they showed that 1-RM performance is influenced not only by maximal force capacity (F_0_), but also by velocity characteristics (V_0_) and the individual F–V slope. Specifically, the difference between F_0_ and the mean force at 1-RM was strongly associated with V_0_ (r = 0.78), indicating that athletes with higher V_0_ are able to express a force at 1-RM that is closer to their theoretical maximal force. Accordingly, although F_0_ values were comparable between groups in the present study, the significantly higher V_0_ observed in high jumpers may have allowed them to express greater force at a velocity corresponding to 1-RM, thereby contributing to their superior maximal strength and competitive performance.

Another finding of the present study was that scaling methodology influences the interpretation of strength–performance relationships. While relative strength (kg∙kg^−1^) showed moderate associations, allometric normalization strengthened the observed relationships between strength derivatives and total performance. Previous research suggested that the scaling approach may vary depending on sample characteristics and sex distribution [2]. Therefore, the present findings should be interpreted within the context of the relatively homogeneous group of weightlifters, where allometric adjustment appeared to provide a slightly stronger representation of the strength–performance relationship.

One limitation of the present study is the cross-sectional design, which precludes causal inference. The sample size was modest (*n* = 14), which increases uncertainty in correlation estimates and may inflate sampling variability. In addition, multiple bivariate correlations were examined, which increases the risk of Type I error; therefore, the consistency of patterns across outcomes (rather than isolated *p*-values) should be prioritized in interpretation. Moreover, F–V profiling was derived from smartphone-based jump assessment under external loads. Although validated procedures were used, measurement noise and technique variability under higher loads may influence the estimation of individual F–V parameters. In addition, the subgroup comparison based on a median split of CMJ height was exploratory in nature. Therefore, these between-group findings should be interpreted as a practical comparison within the present sample rather than as evidence of a definitive athlete classification approach. Furthermore, no multivariable or partial-correlation analyses were performed. Therefore, the present findings should not be interpreted as demonstrating independent associations of force–velocity characteristics beyond maximal strength or body mass.

## 5. Conclusions

The present study demonstrates that maximal dynamic strength—especially 1-RM clean—is strongly associated with competitive Olympic weightlifting performance in trained weightlifters. These findings support the important role of lower-body multi-joint strength capacity as a primary factor associated with competitive outcomes. In addition, velocity- and power-oriented force–velocity characteristics (e.g., V_0_ and Pmax) were also associated with performance, whereas theoretical maximal force (F_0_) showed no meaningful associations within this relatively homogeneous group of weightlifters. Between-group comparisons further indicated that athletes with greater jump performance exhibited higher maximal strength, greater V_0_, and higher power output, despite comparable anthropometric characteristics and similar F_0_ values. Collectively, these findings suggest that, among already strength-trained athletes, the ability to express force at higher contraction velocities may be associated with differences in competitive performance. Additionally, the magnitude of strength–performance relationships was influenced by the scaling approach applied, with allometric normalization slightly strengthening associations compared to simple ratio scaling. This highlights the importance of carefully selecting normalization strategies when examining performance-related variables in strength–power sports. Overall, maximal strength appears to represent an important prerequisite for competitive weightlifting performance; however, velocity-oriented neuromuscular characteristics may also be relevant in explaining performance differences among athletes with similar strength levels. Accordingly, integrating force–velocity profiling into athlete monitoring may provide useful complementary information for identifying differences in velocity and power characteristics that are associated with competitive performance.

## Figures and Tables

**Table 1:** Descriptive characteristics of the sample. Values are presented as mean ± SD.

Variable	Mean ± SD	Min–Max
Age (years)	27.6 ± 4.2	20–35
Body mass (kg)	85.1 ± 6.7	75–96.5
Body fat (%)	11.7 ± 2.8	7–16.5
Snatch (kg)	116.5 ± 13.1	95–140
Clean & Jerk (kg)	146.6 ± 17.1	116–170
Total (kg)	263.2 ± 28.5	211–305
Sinclair (a.u.)	330.4 ± 32.0	286–375
Front squat 1-RM (kg)	166.4 ± 16.6	130–190
Clean 1-RM (kg)	145.3 ± 15.2	120–170
CMJ (cm)	40.3 ± 4.7	33.4–50.9
F_0_ (N)	3606 ± 175	3262–3900
V_0_ (m·s^−1^)	2.82 ± 0.34	2.24–3.40
Pmax (W)	2540 ± 332	1935–3315
Pmax (W·kg^−1^)	29.9 ± 3.3	25.6–37.0

**Table 3:** Pearson Correlations Between Front Squat, Clean Performance, and Lower-Limb Force–Velocity Profile Parameters.

Variable	FS	Clean
FS	**-**	0.86 **
F_0_	0.19	0.43
V_0_	0.64 *	0.62 *
Pmax (W)	0.65 *	0.72 **
Pmax (W∙kg^−1^)	0.65 *	0.54 *
Pmax (W∙kg^−0.67^)	0.68 **	0.64 **
CMJ	0.67 **	0.59 *

**: *p* < 0.01; *: *p* ≤ 0.05.

**Table 4:** Between-Group Comparisons Based on CMJ Performance (High vs. Moderate Jumpers).

Variable	High Jumpers (*n* = 7)	Moderate Jumpers (*n* = 7)	t-Value	*p*	Hedges’ g (95% CI)
Height (cm)	173.1 ± 4.9	175.7 ± 5.3	0.36	0.841	0.48 (−3.1 to 2.2)
Body mass (kg)	81.76 ± 7.32	84.96 ± 7.32	0.50	0.506	0.41 (−4.2 to 3.4)
Body fat (%)	12.0 ± 2.6	11.3 ± 3.1	0.64	0.709	0.23 (−1.3 to 1.7)
Snatch (kg)	124.5 ± 11.6	108.6 ± 9.6	2.79	0.016 *	**1.40** (−4.2 to 7.0)
Clean & Jerk (kg)	155.7 ± 16.6	137.6 ± 12.9	2.28	0.041 *	**1.14** (−6.7 to 8.9)
Total (kg)	280.2 ± 26.4	246.2 ± 19.6	2.74	0.018 *	**1.37** (−10.8 to 13.5)
Sinclair	354.4 ± 19.1	306.5 ± 22.8	4.26	0.001 **	**2.13** (−8.9 to 13.1)
1-RM Front Squat (kg)	178.6 ± 10.1	154.1 ± 12.0	4.11	0.001 **	**2.06** (−3.7 to 7.9)
1-RM Clean (kg)	153.3 ± 13.7	137.4 ± 12.9	2.23	0.045 *	**1.12** (−5.9 to 8.9)
F_0_ (N)	3585 ± 187	3627 ± 172	0.44	0.67	0.21 (−3.1 to 2.2)
F_0_ (N·kg^−1^)	43.2 ± 3.4	42.0 ± 3.1	0.67	0.51	0.34 (−94 to 93)
V_0_ (m·s^−1^)	3.02 ± 0.30	2.61 ± 0.23	2.87	0.014 *	**1.44** (1.3 to 1.6)
Pmax (W·kg^−1^)	32.44 ± 2.65	27.28 ± 1.06	4.79	0.001 **	**2.40** (1.3 to 3.5)
Pmax (W·kg^−^^0.67^)	139.7 ± 12.4	118.9 ± 7.2	3.82	0.002 **	**1.92** (−4.2 to 5.6)
CMJ (cm)	44.0 ± 3.6	36.6 ± 1.8	4.82	0.0001 **	**2.43** (0.9 to 3.9)

**: *p* < 0.01; *: *p* ≤ 0.05.

## Data Availability

The data are available upon request to the corresponding author.
